# Alcohol use patterns and risk of incident cataract surgery: a large scale case–control study in Japan

**DOI:** 10.1038/s41598-022-24465-2

**Published:** 2022-11-22

**Authors:** Kota Fukai, Ryo Terauchi, Yuko Furuya, Kei Sano, Shoko Nakazawa, Noriko Kojimahara, Keika Hoshi, Tadashi Nakano, Akihiro Toyota, Masayuki Tatemichi

**Affiliations:** 1 Department of Preventive Medicine, Tokai University School of Medicine, Isehara, Japan; 2 Department of Ophthalmology, The Jikei University School of Medicine, Tokyo, Japan; 3 Department of Public Health, Shizuoka Graduate University of Public Health, Shizuoka, Japan; 4 Center for Public Health Informatics, National Institute of Public Health, Wako, Japan; 5 Department of Hygiene, School of Medicine, Kitasato University, Sagamihara, Japan; 6 Chugoku Rosai Hospital Research Center for the Promotion of Health and Employment Support, Japan Organization of Occupational Health and Safety, Hiroshima, Japan

**Keywords:** Lens diseases, Epidemiology, Risk factors

## Abstract

To examine the risk of incident cataract surgery associated with alcohol use patterns among Japanese adults. This was a case–control study evaluating 14,861 patients with incident cataract surgery and 14,861 matched controls. Subjects admitted to any of the 34 hospitals in Japan and aged between 40 and 69 years were included. Drinking patterns (drinking frequency, daily average drinks, and total amount of lifetime drinking), smoking history, lifestyle-related comorbidities, and occupational factors were surveyed by trained interviewers. Odds ratios (ORs) and 95% confidence intervals (CIs) were estimated using conditional logistic regression models. For drinking frequency, ORs in the 1–3 days/week and 4–7 days/week groups were 1.10 (95% CI 1.03–1.17) and 1.30 (1.21–1.40), respectively. For average drinks, ORs in > 0–2 drinks/day, > 2–4 drinks/day, and > 4 drinks/day were 1.13 (1.06–1.20), 1.23 (1.12–1.35), and 1.16 (1.03–1.31), respectively. Both men and women had an increased risk of incident cataract surgery with increased total lifetime drinking, with a significant increase in risk occurring at > 90 drink-years for men and > 40 drink-years for women. A positive dose–response relationship was observed between alcohol consumption and cataract. Restricted drinking may help to reduce the progression of cataracts.

## Introduction

Age-related cataract (ARC) is the leading cause of visual impairment and blindness worldwide^1^. In 2020, ARC reportedly caused 78.8 million cases of moderate and severe visual impairment and 15.2 million cases of blindness^1^. With an ageing global population, the incidence of ARC is on the rise. Visual impairment due to ARC can be avoided via treatment using surgical lens extraction^2^. However, not all patients with ARC are eligible for surgery due to physical factors that make surgery intolerable or socioeconomic factors such as medical costs^3^. Although ageing is the most significant risk factor for ARC, and the number of patients is predicted to rise as the population ages, understanding modifiable risk factors will enable the development of preventive measures that will lower the incidence and visual impairment caused by ARC.

Several studies have investigated the associations between alcohol consumption and ARC; however, the results have been inconsistent. Two meta-analyses have been reported on the association between alcohol use and ARC^4,5^. Despite the fact that these meta-analyses reported a significantly increased risk for heavy drinkers compared to that of non-drinkers, the risks differ between studies, with cohorts and case–control studies concluding no associations^6–11^. Studies have reported that the risk of light to moderate drinking is not associated with ARC; conversely, they have a protective effect^12^. In Asian populations, the relationship remains controversial, with positive^13^, inverse^14,15^, and no associations^16–18^ being reported between ARC and alcohol use, as in the studies in Western countries described above. These inconsistencies are attributed to various factors, such as the method of exposure assessment of alcohol consumption, the definition of ARC, or the selection of the target population^19^. Above all, because of the limited number of studies that examined the association between ARC and alcohol consumption, studies in different ethnicities remain warranted^4^. To the best of our knowledge, there are no reports on the association between ARC or incident cataract surgery and alcohol use in Japan, which is the most super-ageing society in the world.

In this large retrospective observational study, we examined the association between alcohol use patterns and incident cataract surgery using the data of the Inpatient Clinico-Occupational Database of Rosai Hospital Group (ICOD-R), a nationwide multicentre hospital-based inpatient registry database in Japan. We conducted a detailed survey on alcohol use patterns, including drinking frequency, daily average drinks, and total amount of lifetime drinking, and examined the associations of other modifiable confounders such as smoking, occupational radiation exposure, outdoor work experience, and lifestyle-related comorbidities (hypertension, diabetes, hyperlipidemia, and obesity). Consistency across different populations is crucial to consider epidemiological causal relationships. The purpose of this study was to examine the relationship between alcohol consumption and incident cataract surgery, which has not been reported in the Japanese population, using the large ICOD-R database. We examined multiple exposure assessment methods with respect to alcohol consumption while examining a variety of confounders.

## Materials and methods

### Study setting and population

The ICOD-R is an ongoing large-scale survey (around 250,000 admissions per year from 34 regional core hospitals all over Japan) conducted by the Japan Organization of Occupational Health and Safety (JOHAS), as described elsewhere^20–24^. ICOD-R consists of approximately 4 million hospitalisation records collected since 1984 by one of the largest hospital groups in Japan, the Rosai Hospital Group, making it the largest inpatient database in Japan. In brief, ICOD-R aims to investigate the link between lifestyle and occupational risk factors and disease prevention.

The clinical diagnosis and surgical procedures were coded according to the International Statistical Classification of Diseases and Related Health Problems, 9th and 10th Revisions (ICD-9 and ICD-10). The ICOD-R is unique to the JOHAS and differs from medical claims data, which improves diagnostic accuracy. Patient profiles of clinical diagnosis were nationally representative, as reported in previous studies^25,26^. Lifestyle-related factors, such as alcohol use, smoking habits, and occupational history information was obtained through interviews based on a formatted questionnaire administered by trained occupational history surveyors at each hospital. All information in the ICOD-R was registered by the health information manager assigned to each hospital. Written informed consent was obtained from all participants. The study adhered to the tenets of the Declaration of Helsinki. The study conformed to the Strengthening the Reporting of Observational Studies in Epidemiology (STROBE) checklist, which is used to improve the quality of reporting of observational studies^27^. Access to the dataset was granted by a research agreement between the JOHAS and researchers. This study was approved by the Research Ethics Committee of JOHAS (approval no. R1-006) and Tokai University School of Medicine (Approval no. 18R-309).

The inclusion criteria for this study were participants in ICOD-R aged 40–69 years at the date of admission between 1 April 2005 and 31 March 2020, including 1,270,536 hospitalisation records of 661,680 individuals. Because of the higher prevalence of ARC in older age groups, this study limited subjects below the age of 70 years to examine the association of alcohol^28^.

### Cases and controls

The cases were those admitted for the following cataract surgery types: senile incipient cataract (ICD-10, H25.0); senile nuclear cataract (H25.1); senile cataract, Morgagnian type (H25.2); senile cataract, unspecified (H25.9); infantile, juvenile, and presenile cataract (H26.0); or cataract, unspecified (H26.9). Cases were only selected if they were admitted to an ophthalmology department for the first time during the study period. Surgical records were classified into ICD-9 procedure codes, and 99.5% of the cases had records of ‘Phacoemulsification of lens’ or ‘Insertion of prosthetic replacement for lens’. Among the eligible cases, 0.5% had missing data on surgical records but were considered to have been admitted for surgical procedures due to the nature of hospitalisation.

Controls were selected according to the methodology described in previous studies^20,22^. These were patients with the following: infectious and parasitic diseases (A00–B99); diseases of the ear and mastoid process (H60–H95); diseases of the skin and subcutaneous tissue (L00–L99); diseases of the genitourinary system (N00–N99); pregnancy, childbirth, and the puerperium (O00–O99); certain conditions originating in the perinatal period (P00–P96); and factors influencing health status and contact with health services (Z00–Z99). The patients were excluded if they had a previous history of cataract (H25.0, H25.1, H25.2, H26.0, or H26.9), presence of an intraocular lens (Z96.1), or mechanical complications of the intraocular lens (T85.2). One control subject was randomly selected for each case by exact matching by gender (male or female), age (same strata by five years), date of admission (same year), and hospital admission (34 hospitals). None of the controls was matched to a case more than once.

### Assessment of alcohol consumption and other covariates

This study evaluated three alcohol consumption variables: drinking frequency, daily average drinks, and total amount of lifetime drinking. On admission to the hospital, each study participant provided information on their average daily intake of standardized alcohol units and the frequency of their drinking. Years from the beginning of drinking until the end of drinking—or the age at admission if they hadn't stopped—were counted toward the length of drinking. Those who declared that they had never drunk alcohol were considered to be lifetime abstainers. Drinking frequency was categorised into four groups: never, former, 1–3 days/week, and 4–7 days/week. Since the questionnaire was modified between 2005 and 2020, the categories related to the drinking frequency were combined so as not to differ from their actual values. Daily average drinks were calculated into continuous values, standard drinks per day (drinks/day)^29^, where one drink/day is equal to 10 g of daily pure ethanol intake. Then, avarage drinks were categorised into four groups (never, > 0–2, > 2–4, and > 4 drinks/day). Meanwhile, a simple measurement of the lifetime burden from drinking, the total amount of lifetime alcohol consumption, has also been used in clinical settings, particularly for cancer epidemiological studies. Total amount of lifetime drinking was calculated by multiplying the daily average drinks (drinks per day) and the duration of drinking (years), based on the methodology used in a previous study^20^. We then categorised patients into five groups according to their drink-year levels (never, > 0–40, > 40–60, > 60–90, and > 90 drink-years).

Confounding variables included smoking history (never, former, or current); lifestyle-related comorbidities (hypertension, diabetes, hyperlipidaemia, and obesity); occupational radiation exposure (yes, no); and outdoor work (yes, no). Workers in Japan who work with radiation are required by law to undergo special medical examinations, and occupational radiation exposure was defined on the basis of such examinations over the course of their lifetime occupation. Lifetime occupational history information was coded according to the Japan Standard Occupational Classification published by the Japanese Ministry of Internal Affairs and Communications^30^. Based on previous studies, outdoor work was defined as the longest lifetime occupation involving high occupational ultraviolet exposure, including: skilled agricultural, forestry and fishery workers, craft and related trades workers, and plant and machine operators and assemblers^31^.

### Statistical analysis

Between cases and control groups, Chi-square tests were performed for comparisons of categorical variables and t-tests for continuous variables. Odds ratios (ORs) and 95% confidence intervals (CIs) for incident cataract surgery were estimated against the three variables for alcohol use patterns using conditional logistic regression models with multiple imputations. Since our analytic sample had 6.4% of missing data on smoking history, we conducted multiple imputations and generated five imputed datasets for the missing data using the Multiple Imputation by Chained Equations method^32^. For model 1, sex, age, admission date, and hospital admission were adjusted. For model 2, the confounding variables of smoking history, lifestyle-related comorbidities (hypertension, hyperlipidaemia, diabetes, and obesity), occupational radiation exposure, and outdoor work were adjusted. The main analyses were conducted using the total population, and then stratified by sex. Linear trend for the association of alcohol use patterns was tested by treating these variables as continuous. In the sensitivity analysis, the analyses above were examined for patients without diabetes. Additionally, the analyses above were examined by age: 40–59 years and 60–69 years. Alpha was set at 0.05, and all p-values were two-sided. All analyses were performed using the Statistical Analysis System (SAS) Software version 9.4 (SAS Institute, Cary, NC, USA).

## Results

The analytic sample included 29,722 participants (14,861 incident cataract surgery cases and 14,861 matched controls). The baseline characteristics of the cases and controls are shown in Table 1. The control group had a higher proportion of those with no drinking history than that of the case group. The prevalence of diabetes and hypertension was significantly higher in the case group. The case group was more likely to be engaged in outdoor work. There were no significant differences between the groups with regard to the matched and other confounding variables.

**Table 1:** Characteristics of cases and controls.

Characteristics	Controls, N (%)	Cases, N (%)	p-value
Total	14,861 (100)	14,861 (100)	
**Sex**			1.00
Men	7367 (49.6)	7367 (49.6)	
Women	7494 (50.4)	7494 (50.4)	
**Age, years**			1.00
40–49	706 (4.8)	706 (4.8)	
50–59	3008 (20.2)	3008 (20.2)	
60–69	11,147 (75.0)	11,147 (75.0)	
Mean (SD)	62.3 (6.1)	62.5 (6.0)	
**Drinking frequency**			< 0.01
Never	6953 (46.8)	6590 (44.3)	
Former	1296 (8.7)	1227 (8.3)	
1–3 days/week	3636 (24.5)	3657 (24.6)	
4–7 days/week	2976 (20.0)	3387 (22.8)	
**Average drinks**			< 0.01
Never	6953 (46.8)	6590 (44.3)	
> 0–2 drink/day	5641 (38.0)	5790 (39.0)	
> 2–4 drink/day	1538 (10.3)	1698 (11.4)	
> 4 drink/day	729 (4.9)	783 (5.3)	
Mean (SD)	1.4 (1.9)	1.5 (2.0)	< 0.01
**Total amount of lifetime drinking**			< 0.01
Never	6953 (46.8)	6590 (44.3)	
> 0–40 drink–years	1307 (8.8)	1187 (8.0)	
> 40–60 drink–years	1048 (7.1)	1093 (7.4)	
> 60–90 drink–years	2602 (17.5)	2670 (18.0)	
> 90 drink–years	2951 (19.9)	3321 (22.3)	
Mean (SD)	52.2 (74.5)	56.9 (76.9)	< 0.01
**Smoking history**			0.18
Never	6752 (45.4)	7219 (48.6)	
Former	3895 (26.2)	4011 (27.0)	
Current	2660 (17.9)	2915 (19.6)	
Missing data	1554 (10.5)	716 (4.8)	
Hypertension, yes	4302 (28.9)	4559 (30.7)	< 0.01
Diabetes, yes	2032 (13.7)	3608 (24.3)	< 0.01
Hyperlipidaemia, yes	2004 (13.5)	2100 (14.1)	0.11
Obesity, yes	1825 (12.3)	1917 (12.9)	0.11
Occupational radiation exposure, yes	35 (0.2)	40 (0.3)	0.56
Outdoor work, yes	2421 (16.3)	2594 (17.5)	< 0.01
**Years in study**			1.00
2005–2010	6976 (46.9)	6976 (46.9)	
2011–2019	7884 (53.1)	7884 (53.1)	

Data are shown as mean (standard deviation) for continuous variables and number (percentages) for categorical variables. P values were for the *t* test for continuous variables and Chi-square test for categorical variables.

*SD* standard deviation.

**Figure 1:**
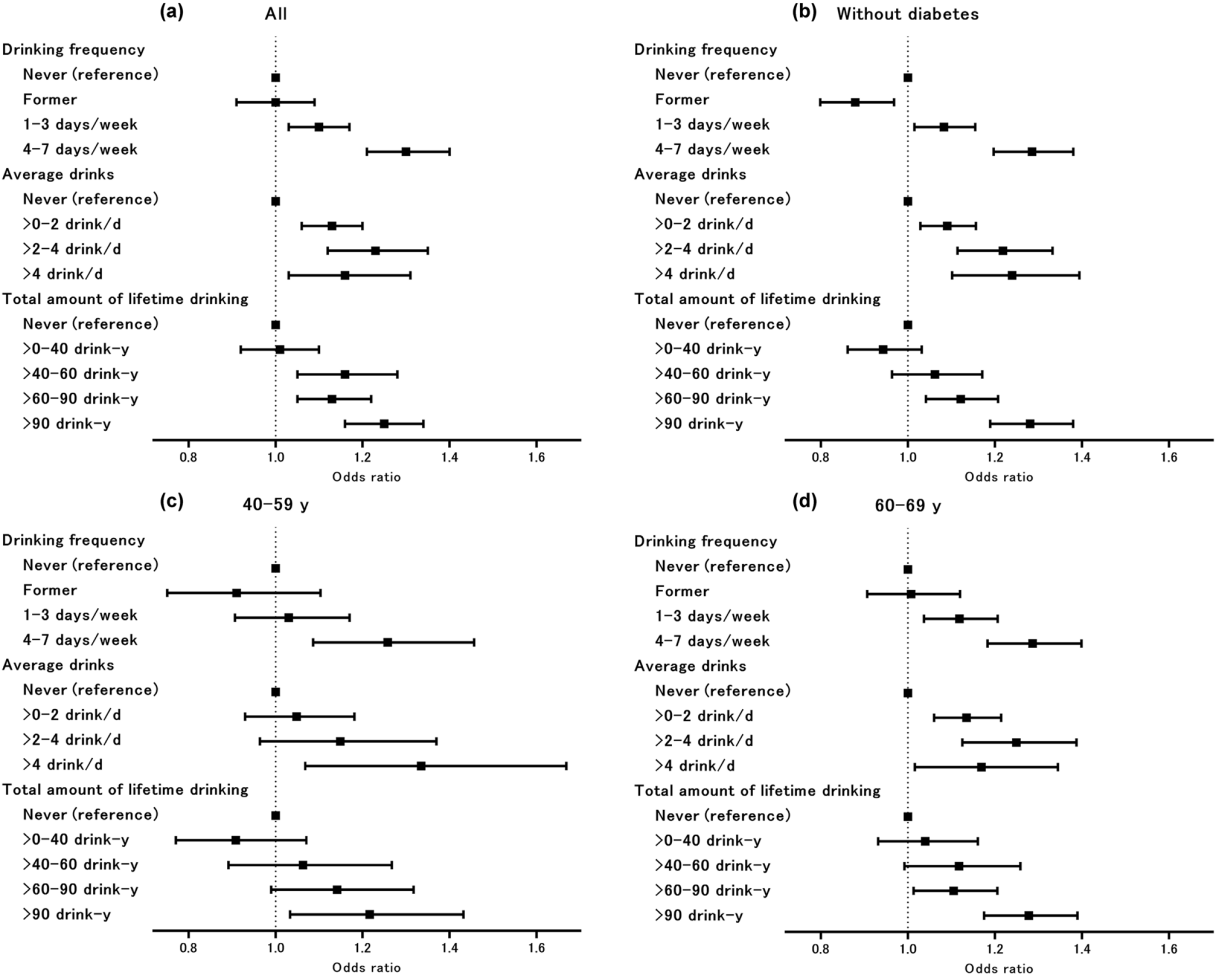
Odds ratios for incident cataract surgery by alcohol use patterns. The odds ratio (dot) and 95% CI (bar) were estimated via conditional logistic regression with multiple imputations, matched for age, sex, admission year, and admitting hospital, and additionally adjusted for smoking history, lifestyle-related comorbidities (hypertension, hyperlipidaemia, diabetes, and obesity), occupational radiation exposure, and outdoor work. (**a**) Conducted among the total population; (**b**) excluded patients with diabetes; (**c**) aged 40–59 years; (**d**) aged 60–69 years.

Table 2 and Fig. 1a show the ORs for incident cataract surgery according to the alcohol use patterns for the total population. Those who drank more frequently, had higher daily average drinks, and higher total amount of lifetime drinking showed a higher risk for incident cataract surgery. These trends of higher ORs for those with higher frequency, average drinks, and total amount of lifetime drinking were robust after adjustment for the confounding variables.

**Table 2:** Odds ratios for incident cataract surgery estimated with the categorical alcohol use patterns (both men and women).

	Controls, N (%)	Cases, N (%)	OR (95% CI)	
			Model 1^b^	Model 2^c^
**Drinking frequency**				
Never	6953 (46.8)	6590 (44.3)	1 (reference)	1 (reference)
Former	1296 (8.7)	1227 (8.3)	1.07 (0.98–1.18)	1.00 (0.91–1.09)
1–3 days/week	3636 (24.5)	3657 (24.6)	1.13 (1.06–1.21)	1.10 (1.03–1.17)
4–7 days/week	2976 (20.0)	3387 (22.8)	1.32 (1.22–1.42)	1.30 (1.21–1.40)
P for trend^a^			< 0.01	< 0.01
**Average drinks**				
Never	6953 (46.8)	6590 (44.3)	1 (reference)	1 (reference)
> 0–2 drink/day	5641 (38.0)	5790 (39.0)	1.16 (1.09–1.23)	1.13 (1.06–1.20)
> 2–4 drink/day	1538 (10.3)	1698 (11.4)	1.27 (1.16–1.39)	1.23 (1.12–1.35)
> 4 drink/day	729 (4.9)	783 (5.3)	1.24 (1.10–1.39)	1.16 (1.03–1.31)
P for trend^a^			< 0.01	< 0.01
**Total amount of lifetime drinking**				
Never	6953 (46.8)	6590 (44.3)	1 (reference)	1 (reference)
> 0–40 drink–years	1307 (8.8)	1187 (8.0)	1.00 (0.91–1.10)	1.01 (0.92–1.10)
> 40–60 drink–years	1048 (7.1)	1093 (7.4)	1.18 (1.07–1.30)	1.16 (1.05–1.28)
> 60–90 drink–years	2602 (17.5)	2670 (18.0)	1.18 (1.09–1.27)	1.13 (1.05–1.22)
> 90 drink–years	2951 (19.9)	3321 (22.3)	1.32 (1.23–1.43)	1.25 (1.16–1.34)
P for trend^a^			< 0.01	< 0.01

*OR* odds ratio, *CI* confidence interval.

^a^Trend test was calculated for the associations between alcohol use patterns as a continuous variable and incident cataract surgery.

^b^Conditional logistic regression matched for sex, age, admission date, and hospital.

^c^Additionally adjusted for smoking history, lifestyle-related comorbidities (hypertension, hyperlipidaemia, diabetes, and obesity), occupational radiation exposure, and outdoor work.

The ORs for incident cataract surgery according to alcohol use patterns by sex are shown in Table 3 (men) and Table 4 (women). Among men, drinking frequency of 4–7 per day/week, average drinks of > 2 drink/day, and total amount of lifetime drinking of > 90 drink-years showed positive associations between incident cataract surgery compared to the control group, after adjusting for confounding variables. Conversely, among women, drinking frequency of 1 or more days per week showed positive associations. For average drinks, only > 0–2 drink/day group showed positive associations, while total amount of lifetime drinking showed positive associations in > 40 drink-year groups.

**Table 3:** Odds ratios for incident cataract surgery estimated with the categorical alcohol use patterns (men).

	Controls, N (%)	Cases, N (%)	OR (95% CI)	
			Model 1^b^	Model 2^c^
**Drinking frequency**				
Never	1948 (26.4)	1948 (26.4)	1 (reference)	1 (reference)
Former	857 (11.6)	857 (11.6)	1.17 (1.03–1.33)	1.05 (0.93–1.19)
1–3 days/week	2056 (27.9)	2056 (27.9)	1.06 (0.95–1.18)	1.01 (0.91–1.12)
4–7 days/week	2506 (34)	2506 (34)	1.32 (1.20–1.47)	1.31 (1.19–1.45)
P for trend^a^			< 0.01	< 0.01
**Average drinks**				
Never	1948 (26.4)	1948 (26.4)	1 (reference)	1 (reference)
> 0–2 drink/day	3448 (46.8)	3448 (46.8)	1.13 (1.03–1.25)	1.09 (0.99–1.20)
> 2–4 drink/day	1333 (18.1)	1333 (18.1)	1.34 (1.20–1.50)	1.29 (1.16–1.45)
> 4 drink/day	638 (8.7)	638 (8.7)	1.29 (1.12–1.48)	1.21 (1.05–1.39)
P for trend^a^			< 0.01	< 0.01
**Total amount of lifetime drinking**				
Never	1948 (26.4)	1948 (26.4)	1 (reference)	1 (reference)
> 0–40 drink–years	543 (7.4)	543 (7.4)	0.93 (0.80–1.09)	0.92 (0.79–1.07)
> 40–60 drink–years	607 (8.2)	607 (8.2)	1.08 (0.93–1.25)	1.07 (0.93–1.24)
> 60–90 drink–years	1736 (23.6)	1736 (23.6)	1.15 (1.03–1.29)	1.11 (0.99–1.23)
> 90 drink–years	2533 (34.4)	2533 (34.4)	1.32 (1.20–1.47)	1.26 (1.14–1.39)
P for trend^a^			< 0.01	< 0.01

*OR* odds ratio, *CI* confidence interval.

^a^Trend test was calculated for the associations between alcohol use patterns as a continuous variable and incident cataract surgery.

^b^Conditional logistic regression matched for sex, age, admission date, and hospital.

^c^Additionally adjusted for smoking history, lifestyle-related comorbidities (hypertension, hyperlipidaemia, diabetes, and obesity), occupational radiation exposure, and outdoor work.

**Table 4:** Odds ratios for incident cataract surgery estimated with the categorical alcohol use patterns (women).

	Controls, N (%)	Cases, N (%)	OR (95% CI)	
			Model 1^b^	Model 2^c^
**Drinking frequency**				
Never	5005 (66.8)	4807 (64.1)	1 (reference)	1 (reference)
Former	439 (5.9)	365 (4.9)	0.89 (0.76–1.03)	0.91 (0.78–1.06)
1–3 days/week	1580 (21.1)	1784 (23.8)	1.22 (1.12–1.33)	1.23 (1.12–1.34)
4–7 days/week	470 (6.3)	538 (7.2)	1.23 (1.07–1.41)	1.34 (1.17–1.54)
P for trend^a^			< 0.01	< 0.01
**Average drinks**				
Never	5005 (66.8)	4807 (64.1)	1 (reference)	1 (reference)
> 0–2 drink/day	2193 (29.3)	2440 (32.6)	1.20 (1.11–1.29)	1.22 (1.13–1.32)
> 2–4 drink/day	205 (2.7)	169 (2.3)	0.89 (0.72–1.10)	0.95 (0.77–1.18)
> 4 drink/day	91 (1.2)	78 (1.0)	0.92 (0.68–1.25)	0.96 (0.70–1.31)
P for trend^a^			0.04	0.04
**Total amount of lifetime drinking**				
Never	5005 (66.8)	4807 (64.1)	1 (reference)	1 (reference)
> 0–40 drink–years	764 (10.2)	748 (10.0)	1.04 (0.93–1.17)	1.08 (0.96–1.21)
> 40–60 drink–years	441 (5.9)	522 (7.0)	1.28 (1.11–1.48)	1.31 (1.14–1.51)
> 60–90 drink–years	866 (11.6)	958 (12.8)	1.21 (1.08–1.35)	1.21 (1.09–1.36)
> 90 drink–years	418 (5.6)	459 (6.1)	1.18 (1.02–1.37)	1.24 (1.07–1.44)
P for trend^a^			< 0.01	< 0.01

*OR* odds ratio, *CI* confidence interval.

^a^Trend test was calculated for the associations between alcohol use patterns as a continuous variable and incident cataract surgery.

^b^Conditional logistic regression matched for sex, age, admission date, and hospital.

^c^Additionally adjusted for smoking history, lifestyle-related comorbidities (hypertension, hyperlipidaemia, diabetes, and obesity), occupational radiation exposure, and outdoor work.

Due to the high prevalence of diabetes in the cases compared to controls, we determined the cataract risk due to alcohol consumption in patients without diabetes in a sensitivity analysis. Figure 1b and Supplementary Table S1 show the ORs of patients without diabetes in the adjusted model. Excluding patients with diabetes from the analyses reduced the risk among former drinkers, but otherwise, the results were similar to the primary findings in relation to alcohol use patterns and incident cataract surgery. Analyses stratified by age under and over 60 years are provided in Fig. 1c,d, respectively. The association between each variable of alcohol use and incident cataract surgery was evident for those over 60 years of age, whereas for those under 60 years of age, the ORs were significantly higher in the group with the highest category of drinking frequency, average drinks, and total amount of lifetime drinking.

## Discussion

In this analysis of Japanese adults aged 40–69 years, we observed positive associations between alcohol consumption habits and incident cataract surgery. We found a dose–response relationship between drinking alcohol and having cataract surgery based on drinking habits. Our study suggests that cataracts may progress even in low frequency and light to moderate drinkers who consume less than 2 drinks (20 g of pure ethanol) per day, compared to that non-drinkers.

Previous epidemiological studies examining the relationship between cataracts and alcohol consumption have reported that moderate alcohol intake contributes to the suppression of cataract^5,12^. A meta-analysis^5^ of five case–control studies on moderate drinkers who consume less than 2 drinks, showed no association with cataract, while heavy drinkers (who consume more than 2 drinks) showed significant association. However, in our population, even small amounts of alcohol had a negative effect on cataract in both men and women. Those previous studies were conducted among Europeans and North Americans, which may explain why our findings differ from theirs. Our findings might be explained by the fact that the Japanese are genetically more susceptible to alcohol toxicity than that other ethnicities. To our knowledge, our study is the first to evaluate this association among a Japanese population. Our results support the view that the patients' drinking history is valuable information to the ophthalmologists when treating ARC and should be routinely collected in clinical settings^4,5,12^.

The results of this study suggest that the associations between drinking habits of men and women and cataract formation differed. Both sexes demonstrated a positive dose–response relationship with drinking frequency; however, women had a higher risk at 1–3 days/week. In terms of average drinks, men in the > 2 drink/day group had a higher risk, whereas women had a higher risk even with a small amount of alcohol consumption. The proportion of women who drank more than 2 drinks/day was small in this study, which might have reduced the reliability of the OR for average drinks among women. Total lifetime drinking did not significantly increase the risk of incident cataract surgery in men with up to 90 drink-years; however, we observed an elevated risk of incident cataract surgery in women with more than 40 drink-years. One explanation may be due, in part, to the differences in alcohol use patterns between men and women^33^. Because women are less likely than males to be habitual drinkers, the risk may have been more apparent for women who drank even a little. Differences in alcohol tolerance between men and women could also be a factor in the gender difference. Despite consuming the same amount of alcohol, women have a smaller volume of distribution of ethanol and a larger area under the blood ethanol concentration–time curve than those of men due to their smaller body size and lower water content^34,35^. In addition to alcohol metabolism, there are also sex differences in alcohol-induced organ damage. For example, women are more sensitive to alcohol-induced liver damage than men^36^.

These results were robust even in our sensitivity analysis, which excluded those with diabetes. In this study, 25% of the patients had diabetes. Patients with diabetes are up to five times more likely to develop cataracts, particularly at an early age^37,38^. Different mechanisms have been proposed to explain cataract promotion in diabetes, including intraocular accumulation of polyols^39–41^ and osmotic and oxidative stress^42^. Because of the strong influence of diabetes mellitus on cataract formation and the possibility that it could not be statistically adjusted for, we performed a sensitivity analysis that excluded patients with diabetes mellitus. The results of the sensitivity analyses and main analyses were consistent.

Interestingly, a reduction in risk was observed among former drinkers when participants with diabetes were excluded, especially among women. It is possible that the reasons for quitting drinking differ between sexes. We believe that, among former drinkers, many men are forced to quit drinking due to health-related reasons, while some women quit drinking due to life changes such as pregnancy and childbirth. Former drinkers are known to have more health problems than those of non-drinkers, but some reports suggest that the results differ between men and women^43^. Furthermore, the decreased risk among former drinkers in our study suggests that light to moderate alcohol consumption can be protective of ocular condition^5,12^. Further epidemiological studies are warranted in the Japanese population in this regard.

Regardless of the results of this study, caution is still needed in understanding the effects of alcohol consumption on incident cataract surgery. Causal interpretation of associational measures estimated in matched case–control studies requires consideration. The matching method used in this study was exact matching. There are other matching methods known as marginal matching, which are based on propensity scores, the probability of receiving exposure within the confounder stratum to which a patient belongs. However, theoretical subtleties are cautioned, such as the lack of justification for interval estimation for propensity score-matched estimates and bias owing to additional adjustment for risk factors not balanced by propensity score matching^44^. In selecting the methodology for this case–control study, given the sufficient sample of controls, we considered it most appropriate to perform exact matching with four well-known demographic factors first, followed by logistic regression analysis to adjust for other confounding factors. Many case–control studies have been reported using this method^20–26^, and we considered that the analytical sample would be representative, both of the cases and controls. The present study adds evidence to previous research on alcohol consumption and incident cataract surgery due to the analysis was conducted with a large sample size, confounding was avoided to the extent possible with the above methodology, and exposure assessment of alcohol consumption was conducted from a variety of perspectives, which, despite being a case–control study, may allow us to get a closer understanding of these relationships.

The mechanism by which alcohol consumption promotes cataract development has not yet been elucidated. The following mechanisms have been assumed in previous reports. Oxidative stress is well known to be involved in the pathogenesis of cataract. Alcohol metabolism is closely related to oxidative stress^45^. Alcohol dehydrogenase is an NAD + dependent enzyme mostly responsible for alcohol metabolism^46^. With the formation of acetaldehyde from alcohol, NAD + is reduced to NADH, which increases the NADH/NAD + ratio in hepatocytes, thereby inducing a redox shift. This redox shift disrupts gluconeogenesis and lipid metabolism in hepatocytes^47^. Another alcohol metabolism pathway is the microsomal ethanol oxidising system, in which the microsomal enzyme cytochrome, CYP2E1, plays an important role^48^. CYP2E1 metabolises alcohol and leads to the production of reactive oxygen species, which cause liver damage and toxicity to other organs^49^. Reactive oxygen species may lead to the aggregation of lens protein, resulting in cataract development^50^. These enzymes might be ectopically expressed in the lens and/or peripheral cells. Furthermore, acetaldehyde, a product of alcohol metabolism, may lead to protein modification with lens opacities^51^. Aldehyde dehydrogenase-2 (ALDH2) plays a role in acetaldehyde detoxification and cytotoxicity inhibition^52^. The ALDH2 gene rs671 polymorphism has a significant effect on enzyme activity, with less than 20% of the enzyme activity remaining in the heterozygous carriers^53^. It is estimated that at least 540 million individuals are ALDH2-deficient worldwide^54,55^, and they are found exclusively in East Asia^56^. Therefore, the Japanese population, with a higher frequency of rs671 polymorphism among East Asians, maybe more strongly affected by acetaldehyde exposure from drinking than that of other ethnicities.

The major strength of this study is that it comprised the largest incident cases than any other epidemiological study examining alcohol consumption and cataracts^4,5^. Moreover, the clinical diagnoses for each patient in the ICOD-R are highly reliable because ophthalmologists completed the clinical information summary with surgical records. In addition, medical histories were completed by physicians based on the ICD-10, and the drinking status was obtained in detail.

However, there are several limitations to our study. First, the selection of hospitalised cases may have introduced a selection bias toward the null. Cataract extraction with intraocular lens implantation is frequently performed as an outpatient procedure. Patients hospitalised for cataract surgery may have had a high risk of complications due to severe lens opacities or poor general condition. We may have underestimated the impact of cataract surgery on patients who are not hospitalised. Second, selection bias by the inclusion of potential hospital controls could have affected the results. However, we conducted our analysis with a variety of controls that were included and excluded based on prior studies, and confirmed not to effect on the results of this study. Third, no information on the types of alcohol was obtained, such as beer, wine, and liqueur. Fourth, since the number of high frequency or heavy drinkers among women was small, the ORs may not have shown a high accuracy.

In conclusion, our findings suggest that alcohol intake is a risk factor for cataract surgery. In the Japanese population, even an infrequent or small amount of drinking may have an adverse association between cataract formation compared to that non-drinking. Since our results suggest that the risk of cataract increases with increased alcohol consumption, indicating a dose–response relationship, it is advised that patients with cataract modify their lifestyle by consuming as little alcohol as possible to reduce the progression of cataract.

## Supplementary Information

Supplementary Information 1.

Supplementary Information 2.

## Data Availability

The datasets used in this study are not publicly available due to restrictions under the license for the current study. These are available on reasonable request from the corresponding author.
