# Supplementary material for: Alcohol use patterns and risk of incident cataract surgery: a large scale case–control study in Japan
Source: Sci Rep. 2022 Nov 22;12:20142. doi: 10.1038/s41598-022-24465-2 (PMC9684480; doi:10.1038/s41598-022-24465-2)
Supplement: Supplementary file 1 — Supplementary Information 1. [file 41598_2022_24465_MOESM1_ESM.docx]

**Supplementary Information**

**Alcohol use patterns and risk of incident cataract surgery: A large scale case-control study in Japan**

Kota Fukai,^1, #^ Ryo Terauchi,^2, #^ Yuko Furuya,^1^ Shoko Nakazawa,^1^ Kei Sano,^2^ Noriko Kojimahara,^3^ Keika Hoshi,^4,5^ Tadashi Nakano,^2^ Akihiro Toyota,^6^ and Masayuki Tatemichi^1*^

^1^Department of Preventive Medicine, Tokai University School of Medicine, Isehara, Japan

^2^Department of Ophthalmology, The Jikei University School of Medicine, Tokyo, Japan

^3^Department of Public Health, Shizuoka Graduate University of Public Health, Shizuoka, Japan

^4^Center for Public Health Informatics, National Institute of Public Health, Wako, Japan

^5^Department of Hygiene, School of Medicine, Kitasato University, Sagamihara, Japan

^6^Chugoku Rosai Hospital Research Center for the Promotion of Health and Employment Support, Japan Organization of Occupational Health and Safety, Hiroshima, Japan

^#^KF and RT contributed equally

***Corresponding author:** Masayuki Tatemichi

Current Affiliation: Department of Preventive Medicine, Tokai University School of Medicine

Telephone number: (+81) 463-93-1121

Email address: tatemichi@tokai-u.jp

**Supplementary Table S1. Sensitivity analysis of the risk of incident cataract surgery on alcohol use patterns among patients without diabetes stratified by sex**

|  | Controls, N (%) | Cases, N (%) | OR (95% CI)^a^ |
| --- | --- | --- | --- |
| Men |  |  |  |
| Drinking frequency |  |  |  |
| Never | 1336 (20.5) | 1118 (17.2) | 1 (reference) |
| Former | 770 (11.8) | 607 (9.3) | 0.93 (0.81 - 1.06) |
| 1-2 days/week | 1719 (26.4) | 1635 (25.1) | 1.13 (1.01 - 1.25) |
| 3-5 days/week | 418 (6.4) | 458 (7.0) | 1.29 (1.10 - 1.52) |
| 6-7 days/week | 2270 (34.9) | 2695 (41.4) | 1.39 (1.25 - 1.54) |
| Average drinks |  |  |  |
| Never | 2622 (40.3) | 2476 (38.0) | 1 (reference) |
| >0–2 drink/day | 2449 (37.6) | 2379 (36.5) | 1.02 (0.93 - 1.13) |
| >2–4 drink/day | 1019 (15.6) | 1146 (17.6) | 1.17 (1.04 - 1.31) |
| >4 drink/day | 423 (6.5) | 512 (7.9) | 1.25 (1.07 - 1.46) |
| Total amount of lifetime drinking |  |  |  |
| Never | 431 (6.6) | 238 (3.7) | 1 (reference) |
| >0–40 drink–years | 2555 (39.2) | 2441 (37.5) | 0.89 (0.74 - 1.07) |
| >40–60 drink–years | 288 (4.4) | 260 (4.0) | 0.97 (0.83 - 1.14) |
| >60–90 drink–years | 395 (6.1) | 379 (5.8) | 1.04 (0.93 - 1.17) |
| >90 drink–years | 1188 (18.2) | 1210 (18.6) | 1.23 (1.11 - 1.36) |
| Women |  |  |  |
| Drinking frequency |  |  |  |
| Never | 3753 (57.3) | 3623 (55.3) | 1 (reference) |
| Former | 426 (6.5) | 325 (5.0) | 0.86 (0.74 - 1.00) |
| 1-2 days/week | 1673 (25.6) | 1806 (27.6) | 1.14 (1.04 - 1.24) |
| 3-5 days/week | 168 (2.6) | 183 (2.8) | 1.19 (0.95 - 1.49) |
| 6-7 days/week | 527 (8.0) | 610 (9.3) | 1.28 (1.12 - 1.46) |
| Average drinks |  |  |  |
| Never | 4452 (68.0) | 4338 (66.3) | 1 (reference) |
| >0–2 drink/day | 1865 (28.5) | 2009 (30.7) | 1.12 (1.03 - 1.22) |
| >2–4 drink/day | 172 (2.6) | 138 (2.1) | 0.90 (0.71 - 1.14) |
| >4 drink/day | 58 (0.9) | 62 (0.9) | 1.22 (0.84 - 1.77) |
| Total amount of lifetime drinking |  |  |  |
| Never | 205 (3.1) | 92 (1.4) | 1 (reference) |
| >0–40 drink–years | 4421 (67.5) | 4333 (66.2) | 1.06 (0.93 - 1.21) |
| >40–60 drink–years | 560 (8.6) | 584 (8.9) | 1.19 (1.02 - 1.39) |
| >60–90 drink–years | 357 (5.5) | 419 (6.4) | 1.15 (1.02 - 1.30) |
| >90 drink–years | 671 (10.2) | 765 (11.7) | 1.12 (0.95 - 1.32) |

^a^ Conditional logistic regression matched for sex, age, admission date, and hospital, additionally adjusted for smoking history, lifestyle-related comorbidities (hypertension, hyperlipidaemia, diabetes, and obesity), occupational radiation exposure, and outdoor work.

OR, odds ratio; CI, confidence interval.
